# Triterpenoid and Coumarin Isolated from *Astilbe grandis* with Anti-Inflammatory Effects through Inhibiting the NF-κB Pathway in LPS-Induced RAW264.7 Cells

**DOI:** 10.3390/molecules28155731

**Published:** 2023-07-28

**Authors:** Jin-Fang Luo, Lan Yue, Tian-Tai Wu, Chen-Liang Zhao, Jiang-Hai Ye, Kang He, Juan Zou

**Affiliations:** 1 School of Pharmacy, Guizhou University of Traditional Chinese Medicine, Guian District, Guiyang 550025, China; luojinfang66666666@163.com (J.-F.L.); wtt5840@163.com (T.-T.W.); zhaochenliang014@gzy.edu.cn (C.-L.Z.); yejianghai013@gzy.edu.cn (J.-H.Y.); 2 School of Basic Medicine, Guizhou University of Traditional Chinese Medicine, Guian District, Guiyang 550025, China

**Keywords:** *Astilbe grandis*, cyclooxygenase 2, lipopolysaccharide, NF-κB, anti-inflammation

## Abstract

The roots of *Astilbe grandis*, known as “Ma sang gou bang”, are used as a Miao traditional medicine with anti-inflammatory and analgesic properties. However, the active components and mechanism of action of this plant remain mostly uncharacterized. The aim of this study was to identify its active components and verify their pharmacological activity. The extract of *A*. *grandis* root was separated using various chromatographic methods. As a result, we obtained one novel triterpenoid, named astigranlactone (**1**), which has an unusual lactone moiety formed between C-7 and C-27. Additionally, a known coumarin compound, 11-*O*-galloyl bergenin (**2**) was isolated from this plant. The structures of these two compounds were elucidated by extensive NMR experiments in conjunction with HR-ESI-MS data. To the best of our knowledge, both compounds were isolated from this species for the first time. Moreover, we tested the anti-inflammation effect of the two compounds by establishing a cellular inflammation model induced by LPS in RAW264.7 cells. The effect of different concentrations of these compounds on the activity of RAW264.7 cells was assessed using a CCK8 assay. The levels of nitric oxide (NO), tumor necrosis factor-α (TNF-α), interleukin-6 (IL-6) and interleukin-1β (IL-1β) in the supernatant of each group were evaluated using the Griess method and an enzyme-linked immunosorbent assay (ELISA). Western blot and quantitative real-time PCR (qRT-RCR) were used to measure the levels of cyclooxygenase 2 (COX-2) and nitric oxide synthase (iNOS) gene expression. Our findings revealed that these two compounds inhibited the high levels of NO, TNF-α, IL-6, IL-1β, COX-2, and iNOS (induced by LPS). Mechanistic studies demonstrated that these two compounds reduced the activation of the nuclear transcription factor-B (NF-κB) signaling pathway by inhibiting the phosphorylation of p65. Therefore, our study indicates that compounds **1** and **2** can exert a definite anti-inflammatory effect by inhibiting the NF-κB signaling pathway.

## 1. Introduction

Inflammation functions as a defensive response to injury stimuli [1]. Within our body system, an appropriate inflammatory response has beneficial effects, such as enhancing damage resistance, clearing infections, and promoting wound healing. However, an excessive inflammatory response can lead to persistent tissue damage and contribute to the development of various diseases, including arthritis [2,3,4], atherosclerosis [5], and others. Furthermore, epidemiological evidence suggests that more than 25% of cancers are attributed to chronic infection or other types of inflammation [6].

Traditional Chinese medicine has a certain advantage in the treatment of inflammatory diseases [7,8,9]. *Astilbe grandis*, belonging to the *Astilbe* genus of the Saxifragaceae family, is known as “Ma sang gou bang” in the Miao nationality areas of China. It has been used in traditional folk medicine to treat inflammation and pain, demonstrating significant anti-inflammatory and analgesic effects [10,11]. Modern research indicates that its congeneric plants exhibit a wide range of biological activities in areas such as analgesia and anti-inflammatory, anti-tumor, cough and asthma relief, anti-bacterial, anti-viral, as well as treatment of burns and scald [12]. However, the bioactive compounds and pharmacological activity of *A. grandis* have received limited research attention.

Our previous phytochemistry investigations on this plant discovered several triterpenes, allyl lactones and other types of compounds [13,14]. In our continuing efforts to identify the active components and verify their pharmacological activity, we conducted a study on its active ingredients and anti-inflammatory properties. In this research, we successfully isolated and identified a novel triterpenoid, named astigranlactone (**1**), which possesses an unusual lactone moiety formed between C-7 and C-27. Additionally, we also isolated a known coumarin, 11-*O*-galloyl bergenin (**2**), for the first time from this species. Furthermore, we performed a preliminary screening experiment to evaluate the anti-inflammatory activity of these compounds. The results showed that both astigranlactone (**1**) and 11-*O*-galloyl bergenin (**2**) reduced the expression levels of inflammatory factors and inflammatory proteins, suggesting a potential anti-inflammatory effect. Finally, our study revealed that these two compounds exerted their definite anti-inflammatory effect by inhibiting the NF-κB signaling pathway. In conclusion, this study successfully identified a novel triterpenoid, astigranlactone (**1**), and a known coumarin, 11-*O*-galloyl bergenin (**2**), from traditional Miao medicine “Ma sang gou bang”. Furthermore, the study conducted a preliminary evaluation of their anti-inflammatory activity and elucidated their mechanism of action. These findings contributed to the scientific understanding of the therapeutic potential and clinical application of “Ma sang gou bang” in the context of anti-inflammatory treatments.

## 2. Results

### 2.1. Identification of Triterpenoid and Coumarin Isolated from A. grandis

Compound **1**, obtained as a colorless needle crystal, was found to have the molecular formula C_32_H_46_O_5_ from its HR-ESI-MS data at *m*/*z* 533.3239 [M + Na]^+^ (calcd 533.3243), corresponding to ten degrees of unsaturation. The IR spectrum of compound **1** showed absorption bands of a carbonyl group (1730 cm^−1^) and an ester carbonyl group (1786 cm^−1^). The ^1^H-NMR spectrum (Table S1) showed signals of an olefinic proton at *δ*_H_ 5.65 (1H, dd, *J* = 5.4, 2.0 Hz, H-12); two oxygenated methine proton at *δ*_H_ 4.40 (1H, dd, *J* = 11.7, 4.1 Hz, H-3) and *δ*_H_ 4.18 (1H, s, H-7); seven tertiary methyl signals at *δ*_H_ 0.84, 0.89, 0.93, 0.95, 0.97, 1.03, 1.29 (3H, each, s, H-28, 30, 23, 29, 25, 26, 24). The ^13^C-NMR and DEPT spectra (Table S1) revealed 32 carbons, including eight methyl carbons, eight methylene carbons, six methine carbons and ten quaternary carbons. A double bond was assigned to the C-12 (*δ*_C_ 127.4) and C-13 (*δ*_C_ 135.0), which was supported by the HMBC correlations (Figure S1) of H-12/C-9 (*δ*_C_ 49.8), C-14 (*δ*_C_ 55.3), C-18 (*δ*_C_ 48.6). Based on this information, compound **1** was assigned as an olean-12-ene-type triterpenoid.

One acetoxy group attached at the C-3 (*δ*_C_ 79.5) position was deduced from the ^1^H-NMR [*δ*_H_ 2.04 (3H, s, H-32)] and ^13^C-NMR [*δ*_C_ 21.3 (C-32), 170.8 (C-31)] data in combination with the HMBC [H-32/C-31 and H-3/C-31] experiments. In addition, the HMBC correlations of H-5 (*δ*_H_ 2.80, 1H, s)/C-6 (*δ*_C_ 206.5) and H-7/C-6 proposed that the location of the carbonyl group was at C-6. Besides the nine degrees of unsaturation ascribed to one double bond, three carbonyl groups and five six-membered rings, the remaining one degree of unsaturation required compound **1** to contain a cyclic ring system. A *γ*-lactone moiety assigned at positions C-7 and C-27 was deduced from the ^1^H-NMR [*δ*_H_ 4.18 (1H, s, H-7)] and ^13^C-NMR [*δ*_C_ 86.1 (C-7), 50.9 (C-8), 55.3 (C-14), 176.7 (C-27)] data, which was also supported by the HMBC correlations of H-7/C-6, C-5 (*δ*_C_ 59.7), C-9, C-14, C-27 and H-15 (*δ*_H_ 1.48 and 1.82, 2H, m)/C-27.

**Figure 1:**
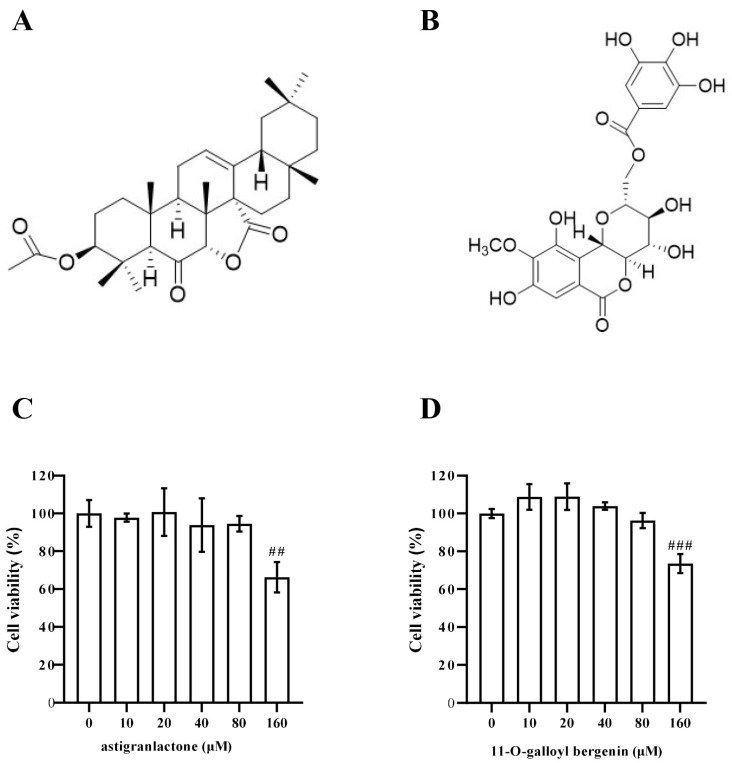
Chemical structural formula of astigranlactone and 11-*O*-galloyl bergenin and their effects on the cell viability of RAW264.7 cells. (**A**) The chemical structural formula of the astigranlactone. (**B**) The chemical structural formula of the 11-*O*-galloyl bergenin. (**C**) The effect of astigranlactone on the cell viability of RAW264.7 cells. (**D**) The effect of 11-*O*-galloyl bergenin on the cell viability of RAW264.7 cells. Results are expressed as the mean value ± SD. ## *p* < 0.01, ### *p* < 0.001 indicates a very significant difference from the normal controls.

In the ROESY spectrum (Figure S7), correlations of H-7 with H-26 (*δ*_H_ 1.03, 3H, s) indicated the *α* orientation of the *γ*-lactone moiety. Compound **1**, 3*β*-acetoxyolean-12-en-27-oic acid [15] and 3*β*, 6*β*-dihydroxyurs-12-en-7*α*,27*α*-olide [16] exhibited the same 3*β* moiety, which was supported by the similar NMR data found for both A rings of them. Consequently, the structure and relative configuration of compound **1** was determined as 3*β*-acetoxyolean-12-en-6-oxo-7*α*,27*α*-olide, which was named astigranlactone (Figure 1).

By comparison of our spectroscopic data with that reported in the literature [17], the known compound **2** (Figure 1) was identified as 11-*O*-galloyl bergenin.

### 2.2. Effect of Astigranlactone and 11-O-Galloyl Bergenin on the Cell Viability of RAW264.7 Cells

The structural formula of the pharmaceutical chemistry of astigranlactone and 11-*O*-galloyl bergenin are shown in Figure 1A,B. As shown in Figure 1C,D, the effect of astigranlactone and 11-*O*-galloyl bergenin on the cell viability of RAW264.7 cells was detected by a CCK8 kit. The experimental results showed that both compounds (below a concentration of 160 µM) did not significantly inhibited cell proliferation when compared with the normal group. The results showed that there were no significant cytotoxic effects of astigranlactone and 11-*O*-galloyl bergenin below 160 µM. Therefore, we chose the concentrations of 20 µM, 40 µM, and 80 µM of these two compounds for further study.

### 2.3. Effect of the Astigranlactone and 11-O-Galloyl Bergenin on NO Secretion in the Supernatant of RAW264.7 Cells Induced by LPS

**Figure 2:**
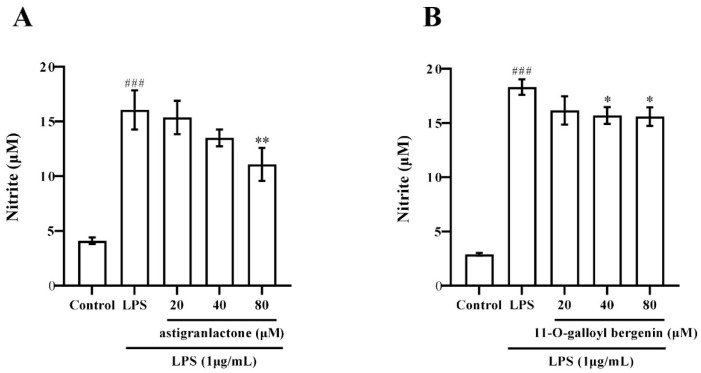
The pharmacological action of astigranlactone and 11-*O*-galloyl bergenin on the levels of NO in LPS-induced RAW264.7 cell supernatant. (**A**) Amount of NO secretion in RAW264.7 cells induced by LPS after pretreatment with astigranlactone. (**B**) Amount of NO secretion in RAW264.7 cells induced by LPS after pretreatment with 11-*O*-galloyl bergenin. Results are expressed as the mean value ± SD. ### *p* < 0.001 indicates a very significant difference from the normal group. * p < 0.05, ** p < 0.01, indicates a significant difference from the model group.

It was found that both astigranlactone and 11-*O*-galloyl bergenin inhibited NO secretion in LPS-induced RAW264.7 cells. As shown in Figure 2A,B, LPS induced a high level of NO in the supernatant of RAW264.7 cells, which was significantly different from the normal group (*p* < 0.001). When the astigranlactone concentration was 80 µM, the secretion of NO in the cells was significantly decreased compared with LPS-induced cells (*p* < 0.01). When the concentration of 11-*O*-galloyl bergenin was 40 µM and 80 µM, the secretion of NO in the cells’ supernatant was reduced when pretreated with 11-*O*-galloyl bergenin, which was significantly different from the model group (*p* < 0.05).

### 2.4. Effect of the Astigranlactone and 11-O-Galloyl Bergenin on the Content of TNF-α, IL-6 and IL-1β in the Supernatant of RAW264.7 Cells Induced by LPS

**Figure 3:**
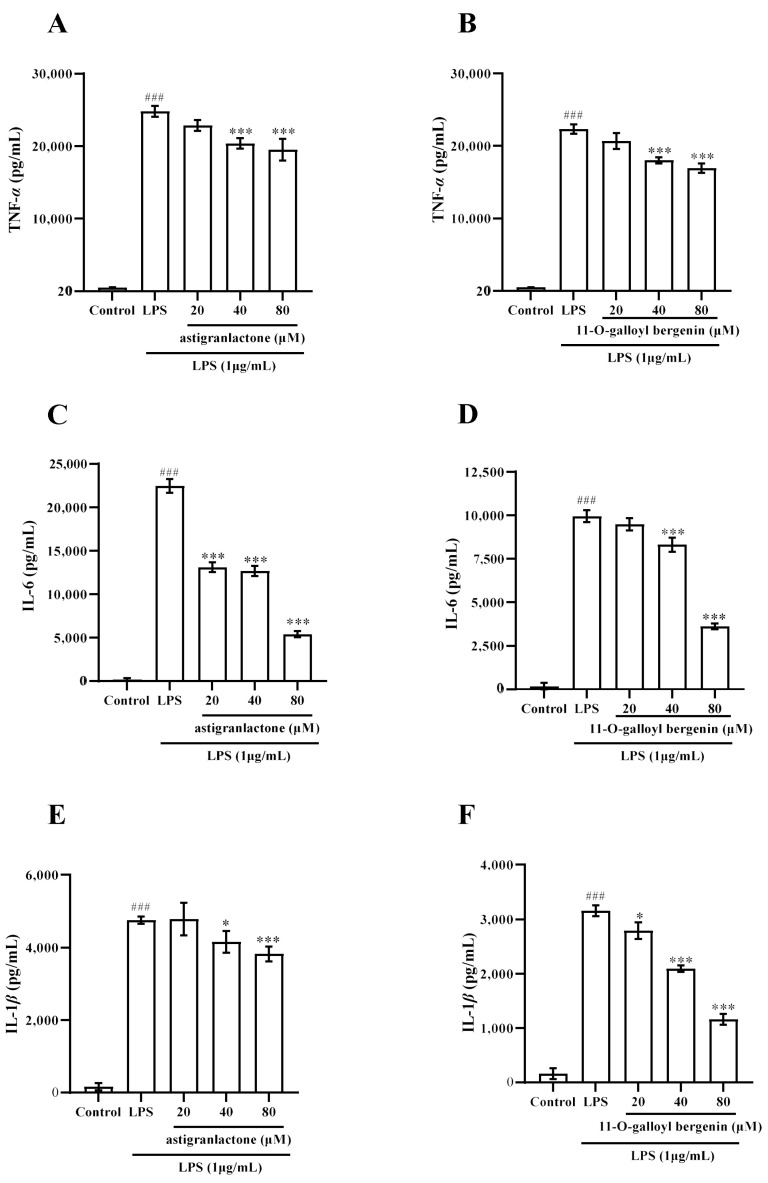
The pharmacological action of astigranlactone and 11-*O*-galloyl bergenin on the levels of TNF-α, IL-6, and IL-1β in LPS-induced RAW264.7 cells. (**A**) Amount of TNF-α in the cell supernatant pretreated with astigranlactone. (**B**) Amount of TNF-α in the cell supernatant pretreated with 11-*O*-galloyl bergenin. (**C**) Amount of IL-6 in the cell supernatant pretreated with astigranlactone. (**D**) Amount of IL-6 in the cell supernatant pretreated with 11-*O*-galloyl bergenin. (**E**) IL-1 β secretion in the cell supernatant pretreated with astigranlactone. (**F**) Amount of IL-1 β in the cell supernatant pretreated with 11-*O*-galloyl bergenin. Results are expressed as the mean value ± SD. ### *p* < 0.001 indicates a very significant difference from the normal group. * *p* < 0.05, *** *p* < 0.001, indicating a significant and very significant difference from the model group.

The experimental results showed that the content of TNF-α, IL-6, and IL-1β were significantly increased in the supernatant of RAW264.7 cells induced by LPS; however, the secretion of these inflammatory factors was reduced when pretreated with astigranlactone and 11-*O*-galloyl bergenin (Figure 3A–F). As shown in Figure 3A–F, the amount of each inflammatory factor in the supernatant of RAW264.7 cells induced by LPS was significantly different from that of the normal group (*p* < 0.001). When the concentrations of astigranlactone and 11-*O*-galloyl bergenin were 40 µM and 80 µM, the amount of TNF-α was significantly reduced (Figure 3A,B), which was significantly different from the model group (*p* < 0.001). When the concentration of astigranlactone was 20–80 µM and the concentration of 11-*O*-galloyl bergenin was 40 µM and 80 µM, the amount of IL-6 in the cell supernatant was significantly reduced (Figure 3C,D), significantly different from the model group (*p* < 0.001). When astigranlactone was at 40 µM and 11-*O*-galloyl bergenin at 20 µM, both compounds reduced IL-1β production in the cell supernatant (Figure 3E,F), which was significantly different from the model group (*p* < 0.05). When the astigranlactone was 80 µM and 11-*O*-galloyl bergenin was 40–80 µM, the IL-1β in the cells’ supernatant was significantly reduced, which was significantly different from the model group (*p* < 0.001).

### 2.5. Effect of the Astigranlactone and 11-O-Galloyl Bergenin on COX-2 and iNOS Expression in LPS-Induced RAW264.7 Cells

**Figure 4:**
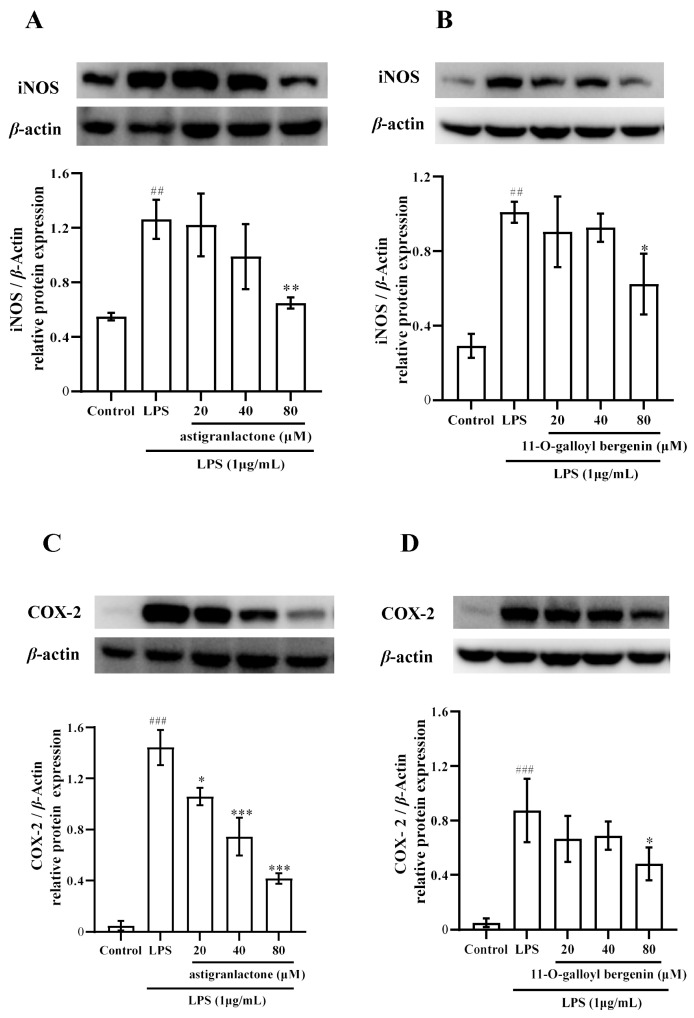
The pharmacological action of astigranlactone and 11-*O*-galloyl bergenin on the levels of iNOS and COX-2 in LPS-induced RAW264.7 cells. (**A**) Expression of iNOS in LPS-induced RAW264.7 cells pretreated with astigranlactone. (**B**) Expression of iNOS in LPS-induced RAW264.7 cells pretreated with astigranlactone. (**C**) COX-2 expression in LPS-induced RAW264.7 cells pretreated with 11-*O*-galloyl bergenin. (**D**) Expression of COX-2 in-LPS induced RAW264.7 cells pretreated with 11-*O*-galloyl bergenin. Results are expressed as the mean value ± SD. ## *p* < 0.01, ### *p* < 0.001 indicates a significant difference from the normal group. * *p* < 0.05, ** *p* < 0.01, *** *p* < 0.001, indicating a significant difference from the model group.

After astigranlactone treatment, the iNOS expression (Figure 4A) in the 80 µM group and the COX-2 expression (Figure 4C) was significantly different from the model group (*p* < 0.001). After 11-*O*-galloyl bergenin treatment, the expression of iNOS (Figure 4B) and COX-2 (Figure 4D) in the 80 µM group was significantly different from the model group (*p* < 0.001).

### 2.6. Effects of Astigranlactone and 11-O-Galloyl Bergenin on the NF-κB Inflammatory Signaling Pathway in LPS-Induced RAW264.7 Cells

**Figure 5:**
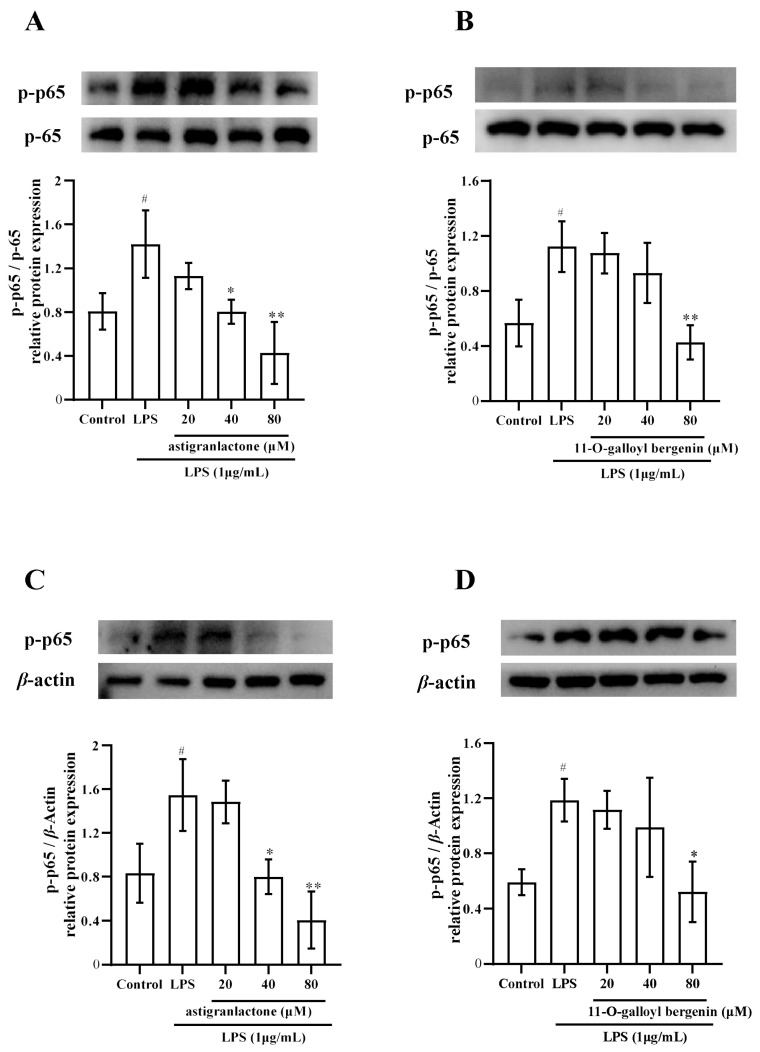
The pharmacological action of astigranlactone and 11-*O*-galloyl bergenin on the levels of p-p65 in LPS-induced RAW264.7 cells. (**A**) Expression of p-p65 (p-p65/p65) in the cells after astigranlactone pretreatment. (**B**) Expression of p-p65 (p-p65/p65) in the cells after pretreatment with 11-*O*-galloyl bergenin (p-p65/β-actin). (**C**) Expression of p-p65 in cells after pretreatment with astigranlactone (p-p65/β-actin). (**D**) Expression of p-p65 in the cells pretreated with 11-*O*-galloyl bergenin (p-p65/β-actin). Results are expressed as the mean value ± SD. # *p* < 0.05, indicating a significant difference from the normal group. * p < 0.05, ** *p* < 0.01, indicating a significant difference from the model group.

The experimental data showed that the expression of phosphorylated p65 protein (Figure 5A,C) in LPS-induced RAW264.7 cells pretreated with astigranlactone was significantly inhibited when compared with the model group (*p* < 0.01), and the expression of phosphorylation of p65 protein (Figure 5B,D) in LPS-induced RAW264.7 cells pretreated with 11-*O*-galloyl bergenin was significantly inhibited when compared with the model group (*p* < 0.01).

### 2.7. Effect of Astigranlactone and 11-O-Galloyl Bergenin on iNOS and COX-2 mRNA Expression in LPS-Induced RAW264.7 Cells

**Figure 6:**
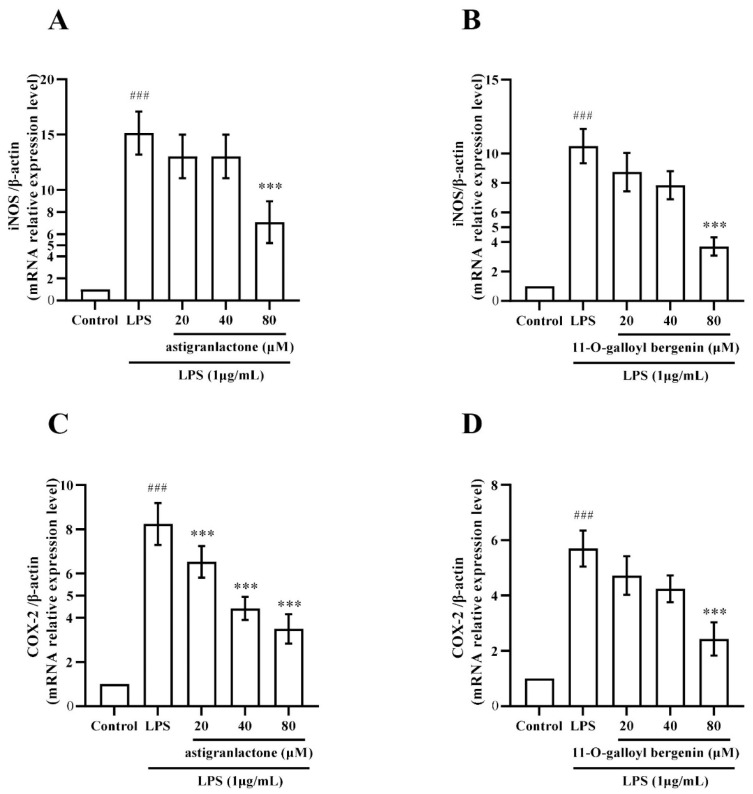
The pharmacological action of astigranlactone and 11-*O*-galloyl bergenin on the levels of iNOS and COX-2 mRNA relative expression in LPS-induced RAW264.7 cells. (**A**) Expression of iNOS mRNA expression in cells after pretreatment with astigranlactone. (**B**) Expression of iNOS mRNA expression in cells pretreated with 11-*O*-galloyl bergenin. (**C**) COX-2 mRNA expression in cells pretreated with astigranlactone. (**D**) Expression of COX-2 mRNA expression in cells pretreated with 11-*O*-galloyl bergenin. Results are expressed as the mean value ± SD. ### *p* < 0.001 indicates a significant difference from the normal group. *** *p* < 0.001, indicating a significant difference from the model group.

RT-PCR was used to detect iNOS and COX-2 mRNA expression. The results showed that iNOS (Figure 6A,B) and COX-2 (Figure 6C,D) were significantly increased in the model group (*p* < 0.001), and astigranlactone and 11-*O*-galloyl bergenin also significantly reduced the expression of iNOS (Figure 6A,B) and COX-2 (Figure 6C,D) mRNA expression, which was significantly different from the model group (*p* < 0.01).

## 3. Discussion

Guizhou Province has abundant resources of Miao medicine [18,19]. However, research on medicinal preparations of Miao medicine in the context of modern medicine is still in its early stages, with only a few of them being thoroughly investigated. Knowledge regarding Miao medicine remains limited, largely based on folklore that Miao medicine can effectively treat certain ailments. We have yet to fully understand its active ingredients and mechanism of action. The exploration of the mechanism underlying Miao medicine remains a challenging yet essential task [2]. Therefore, it is imperative to delve deeper into the treasure trove of Miao medicine.

In this study, novel anti-inflammatory compounds astigranlactone and 11-*O*-galloyl bergenin were isolated from *A*. *grandis*. However, the anti-inflammatory activity and mechanism of these two compounds remain unknown. Therefore, we conducted a preliminary investigation using a classical inflammatory cell model to study the anti-inflammatory effects and mechanisms of these two compounds. The aim of this study was to contribute a new scientific research foundation for the clinical application of Miao medicine “Ma sang gou bang”.

Macrophages, when stimulated, play a role in promoting and worsening inflammation [20,21]. Specifically, during the inflammatory response, macrophages release numerous inflammatory factors and other indicators associated with inflammation, thereby exacerbating the onset and progression of inflammation-related diseases [22,23,24,25]. In this study, it was discovered that astigranlactone and 11-*O*-galloyl bergenin significantly inhibited the production of TNF-α, IL-6 and IL-1β (Figure 3A–F).

LPS induced high expression of inflammatory proteins, and the inhibitory effect on COX2 protein expression is considered to have significant anti-inflammatory effects [25]. In this study, it was found that astigranlactone and 11-*O*-galloyl bergenin significantly suppressed the expression of COX-2 protein (Figure 4C,D).

Our results are in line with recent studies demonstrating that the inhibition of iNOS protein expression can further suppress the production of NO [26,27]. Our findings also reveal that astigranlactone and 11-*O*-galloyl bergenin significantly reduced the elevated levels of NO and iNOS in LPS-induced RAW264.7 cells (Figure 2A,B and Figure 4A,B).

Numerous studies have demonstrated that inhibiting the activation of the NF-κB pathway can effectively decrease the production of downstream inflammatory indicators, thereby exerting an anti-inflammatory effect [28,29,30]. In line with these findings, our study revealed that astigranlactone and 11-*O*-galloyl bergenin significantly inhibited the production of inflammatory mediators, which included NO, TNF-α, IL-6 and IL-1β (Figure 2 and Figure 3).

Previous research has confirmed [29,31,32] that LPS activates the NF-κB pathway, and inhibiting this pathway effectively reduces the release of inflammatory cytokines and the expression of inflammatory proteins [29], thus exerting an anti-inflammatory role. iNOS and COX-2 are downstream indicators of the NF-κB pathway [33,34]. Our study found that astigranlactone and 11-*O*-galloyl bergenin effectively inhibited the expression of iNOS and COX-2 (both at the protein and mRNA levels), thereby promoting their anti-inflammatory activity (Figure 4 and Figure 6).

The inhibitory effect on the NF-κB pathway can significantly suppress the secretion of inflammatory indicators [35,36,37]; the inhibitory effect on the NF-κB pathways is considered an effective approach to control the occurrence and development of inflammatory diseases [38].

In this study, LPS stimulation increased the levels of inflammatory mediators (NO, TNF-α, IL-6 and IL-1β) and inflammatory proteins (iNOS and COX-2). Meanwhile, astigranlactone and 11-*O*-galloyl bergenin significantly suppressed the production of inflammatory cytokines and inflammatory-related proteins induced by LPS. The above results all indicate that astigranlactone and 11-*O*-galloyl bergenin possess notable anti-inflammatory activity.

However, whether astigranlactone and 11-*O*-galloyl bergenin exert this anti-inflammatory effect by inhibiting the activation of the NF-κB pathway remains unknown. Therefore, in the following experiments, we further examined the key signaling pathway protein (p-p65) of the NF-κB pathway to verify whether astigranlactone and 11-*O*-galloyl bergenin exert their anti-inflammatory effect through inhibiting NF-κB pathway activation.

**Figure 7:**
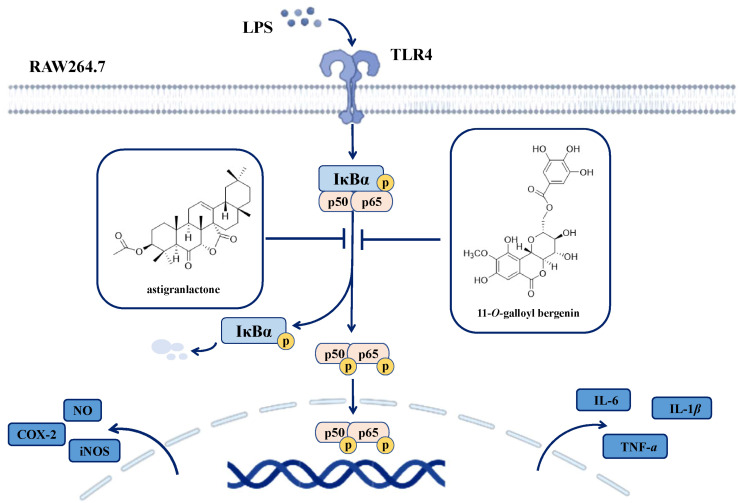
Schematic of the anti-inflammatory activities of astigranlactone and 11-*O*-galloyl bergenin in LPS-induced RAW264.7 cells. Astigranlactone and 11-*O*-galloyl bergenin exert anti-inflammatory activity by inhibiting the expression of inflammatory mediators (NO, TNF-α, IL-6 and IL-1β) and proteins (iNOS and COX-2) via suppression of the NF-κB pathway.

In this study, we demonstrated that LPS stimulation increased the level of p65 phosphorylation. Moreover, pretreatment with astigranlactone and 11-*O*-galloyl bergenin reduced the elevated level of p65 phosphorylation induced by LPS. Astigranlactone and 11-*O*-galloyl bergenin significantly inhibited p65 phosphorylation (Figure 5A–D). In summary, the mechanism diagram (Figure 7) illustrates the mechanism and link between the anti-inflammatory effect of astigranlactone and 11-*O*-galloyl bergenin, providing a preliminary explanation for how these compounds inhibit inflammatory proteins and factors to exert anti-inflammatory effects.

## 4. Materials and Methods

### 4.1. Materials

The RAW264.7 cell line was purchased from Procell Life Science&Technology Co., Ltd. (Wuhan, China). Astigranlactone and 11-*O*-galloyl bergenin were obtained from the Key Laboratory of Traditional Chinese Medicine and Ethnic Medicine, Guizhou University of Traditional Chinese Medicine (Guiyang, China). Fetal calf serum (FBS) and DMEM were from Gibco (Billings, MT, USA). Lipopolysaccharide (LPS) was from Sigma (St. Louis, MO, USA). The NO detection kit was obtained from Beyotime. CCK8 was obtained from TargetMol (Boston, MA, USA). The Mouse TNF-α ELISA Kit, Mouse IL-1β ELISA Kit and Mouse IL-6 ELISA Kit were obtained from MultiSciences Biotech Co., Ltd. (Hangzhou, China). The PAGE gel rapid preparation kit (10%), RIPA cell lysate, BCA Protein Concentration determination kit, 5 protein loading buffer, 10 electrotransfer solution, 5 Trts-glycine electrophoresis buffer and Rainbow 180 broad-spectrum protein Marker were from Solarbio (Beijing, China). TBST buffer, primary antibody dilution and secondary antibody dilution were from Biosharp (Hefei, China). Fast sealing liquid was from Genefist (Abingdon, UK). Luminescent liquid was from Nature Biosciences (New Delhi, India). Antibodies used in the immunoblot analysis, p-p65 and p65 were from Abcam (Cambridge, UK), while COX-2, iNOS and β-actin were from Absin (Shanghai, China). The secondary antibody (Anti-rabbit) was from Solarbio. The RNA extraction kit was from Solarbio. The RNA reverse transcription kit and SYBR Green qPCR Master Mix were from Roche. The primers for RT-PCR were from Huada gene, Guangzhou, China.

### 4.2. Extraction and Isolation of Triterpenoid and Coumarin from A. grandis

The air-dried and powdered roots of *A. grandis* Stapf ex Wils (20 kg) were extracted three times with 90% MeOH at room temperature. The solvent was evaporated under reduced pressure, leaving an extract that was suspended in H_2_O. The suspension was extracted with AcOEt. The AcOEt layer provided a residue which was subjected to silica gel column chromatography (Si CC), and was eluted with a gradient eluent of a CHCl_3_/MeOH solvent system (100:1, 95:5, 9:1, 8:2). Five fractions (Frs. A–E) were collected and subjected to thin-layer chromatography (TLC) analysis. Compound **1** (70 mg) was obtained by further purification of Fr. A (9.7 g) by recrystallization and the use of Sephadex LH-20 (CHCl_3_/CH_3_OH, 1:1). Fraction D (85.4 g) was subjected to a middle chromatogram isolated gel (MCI) column with a gradient eluent of MeOH/H_2_O (3:7, 5:5) to afford fractions E1–E3. Fraction E2 (6.8 g) was subjected to Si CC eluting with a gradient AcOEt/CH_3_OH solvent system (9:1) to afford compound **2** (50 mg).

### 4.3. RAW264.7 Cell Culture

RAW264.7 cells were resuscitated from liquid nitrogen tanks and then subcultured with DMEM complete medium in a 37 °C cell incubator, containing 5% CO_2_, and cells were subcultured three times before use in subsequent experiments. DMEM complete medium contained 1% penicillin/streptomycin (P/S) and 10% FBS, and the cell culture method was the same as in the published literature [39].

### 4.4. Cell Culture and Viability Assay

The effect of two compounds on cell activity was tested by a CCK8 assay according to ref. [40]. Normal cultures of RAW264.7 cells were passaged for more than 3 times, counted, and adjusted to a cell density of 1 × 10^5^ cells/mL, seeded in 100 µL cell suspension per well in 96-well plates and incubated for 20 h in a 37 °C incubator. The concentration gradient of each compound was prepared as 160, 80, 40, 20, 10 and 5 µM. After the culture, the cell supernatant was discarded, and the concentration gradient solution of each compound was added to 100 µL per well, and returned to the 37 °C incubator for 20 h. After the culture, the plates were removed and 10 µL of CCK 8 solution was added to each well and returned into the 37 °C incubator for a further 2 h incubation. The OD at 450 nm was measured using a microplate reader, data was recorded and the relative cell viability was calculated.

### 4.5. Assay of Nitric Oxide Concentration, TNF-α, IL-6, and IL-1β

Inflammatory factors were detected by Griess kit or ELISA kit. RAW 264.7 cells were delivered, counted, adjusted the cell density to 3 × 10^5^ cells/mL, 2 mL/well into 6-well culture plate for 20 h, grouped according to Table 1 and cultured for 20 h. After incubation, the supernatant solution was collected, centrifuged at 1200 rpm for 10 min, and the NO, TNF-*α*, IL-6 and IL-1*β* concentration in the cell supernatant was detected using the NO kit and ELISA kit according to the specification and the published papers [39,40].

### 4.6. Western Blotting to Detect the Expression of COX-2, iNOS, and p-p65

To detect the expression of COX-2, iNOS, and p-p65, normally cultured RAW 264.7 cells were counted, and their cell density adjusted to 5 × 10^5^ cells/mL, followed by 20 h of a 2 mL/well inoculation into 6-well culture plates. To detect the expression of COX-2 and iNOS, the cells were treated with astigranlac-tone or 11-*O*-galloyl bergenin for 1 h, and then incubated with 1 μg/mL LPS for 18 h. To detect the expression of p-p65, the cells were treated with astigranlac-tone or 11-*O*-galloyl bergenin for 1 h, and then incubated with 1 μg/mL LPS for 0.5 h.

Then, total cell protein was collected, quantified and added into the protein loading buffer. The proteins were denatured for Western blotting. Briefly, the experimental procedure for Western blotting is discussed: ① glue making; ② electrophoresis; ③ blocking and incubation; ④ washing, incubation and application of the primary and secondary antibodies. The luminescent liquid was prepared according to the manufacturer’s instructions. this liquid is slowly dropped onto the PVDF film, which is then exposed on the luminometer to collect an image. The relative quantitative analysis of protein expression was carried out using the relative quantitative software Image.

### 4.7. Quantitative Real-Time PCR Method

#### 4.7.1. Total Cellular RNA was Extracted

Treatment: RAW 264.7 cells were delivered, counted, adjusted to a cell density of 5 × 10^5^ cells/mL, 2 mL/well were seeded into 6-well culture plates for 20 h. The experimental groups were performed according to Table 1, treated with different compounds, cultured for 1 h and then incubated with 1 μg/mL LPS for 18 h. After incubation, the cell supernatant was aspirated and discarded, the cells were washed three times with PBS, and used for total RNA extraction.

Total cell RNA extraction was extracted following the instructions of the test kits The obtained total RNA was stored at −80 °C.

#### 4.7.2. RNA Reverse Transcription into cDNA

The prepared RNA described above was removed and, after calculation, the diluted sample was added to the RNA reverse centrifuge tube with 1 µL of Transcriptor Universal cDNA Master 1 and 4 µL of Transcriptor Universal cDNA Master 2. The reaction conditions were set to 25 °C, 5 min; 55 °C, 10 min; 85 °C, 5 min; 16 °C, 30 min (Cycle 1, From 1 to 3). The cDNA was obtained by placing the sample in the PCR instrument.

#### 4.7.3. Quantitative Real-Time PCR

The experiment was performed according to the SYBR Green qPCR Master Mix kit instructions and the published articles [39,40]. The reaction procedure is showed in Table 2.

β-actin was used as the internal reference control for the reaction, the reaction system in Table 3, and the primer sequences for RT-PCR in Table S2.

### 4.8. Statistical Methods

The test results are expressed as mean ± SD through three repeated experiments, and a one-way analysis of variance LSD test using SPSS 19.0 statistical software. *p* < 0.05 was considered to be statistically significant.

## 5. Conclusions

In summary, this study identified a new triterpenoid (astigranlactone) and a known coumarin (11-*O*-galloyl bergenin) from Miao medicine “Ma sang gou bang”. It further demonstrated that astigranlactone and 11-*O*-galloyl bergenin possess potent anti-inflammatory activity in LPS-stimulated RAW264.7 cells via inhibiting of the NF-κB signaling pathway. This inhibitory effect of astigranlactone and 11-*O*-galloyl bergenin results in the suppression of various inflammatory mediators (NO, TNF-α, IL-6 and IL-1β) and inflammatory proteins (iNOS and COX-2). Further mechanism research revealed that astigranlactone and 11-*O*-galloyl bergenin exerted their anti-inflammatory activity through the inhibition of the NF-κB pathway. Overall, this work recommends astigranlactone and 11-*O*-galloyl bergenin as potential novel anti-inflammatory agents, and it suggests that these compounds could be an ideal therapeutic for inhibiting and treating inflammation-related disorders in the future. Furthermore, additional studies are needed to assess the mechanisms underlying the anti-inflammatory efficacy of astigranlactone and 11-*O*-galloyl bergenin using other cell lines and in vivo models.

## Figures and Tables

**Table 1:** Experimental grouping.

Group	Condition of Culture
Control Group	Complete medium was normal normally
Model group	Incubation in complete medium containing LPS (1 µg/mL)
Medication group	Each group with the added corresponding concentration for compounds for 1 h, and then LPS (1 µg/mL)

**Table 2:** Quantitative real-time PCR reaction procedures.

Content	Temperature	Time	Recurring Number
Pre-denaturation	95 °C	10 min	1
Denaturation	95 °C	15 s	40
Annealing extension	60 °C	60 s	

**Table 3:** Quantitative real-time PCR reaction system.

cDNA	4 μL
Forward Primer (10 μM)	0.4 μL
Reverse Primer (10 μM)	0.4 μL
SYBR Green Master Mix	10 μL
50× ROX Reference Dye 2	0.4 μL
H_2_O	4.8 μL

## Data Availability

All data generated or analyzed during this study are included in this published article.

## Supplementary Materials

The following supporting information can be downloaded at: https://www.mdpi.com/article/10.3390/molecules28155731/s1, Table S1: ^1^H-NMR and ^13^C-NMR data of astigranlactone. Table S2: The primer sequences for RT-PCR. Figure S1: HMBC and ROESY correlations of astigranlactone. Figures S2–S9: ^1^H-NMR, ^13^C-NMR and DEPT, HMQC, HMBC, COSY, ROESY, HR-ESI-MS and IR spectrum of astigranlactone.
