# Supplementary material for: Triterpenoid and Coumarin Isolated from Astilbe grandis with Anti-Inflammatory Effects through Inhibiting the NF-κB Pathway in LPS-Induced RAW264.7 Cells
Source: Molecules. 2023 Jul 28;28(15):5731. doi: 10.3390/molecules28155731 (PMC10421095; doi:10.3390/molecules28155731)
Supplement: Supplementary file 1 [file molecules-28-05731-s001.zip › molecules-2504063-supplementary.pdf]

molecules

MDPI

Supplementary Materials

Triterpenoid and Coumarin Isolated from *Astilbe grandis* with Anti-Inflammatory Effects through Inhibiting the NF-κB Pathway in LPS-Induced RAW264.7 Cells

Jin-Fang Luo [1,2,†], Lan Yue [1,†], Tian-Tai Wu [1], Chen-Liang Zhao [1], Jiang-Hai Ye [1], Kang He [1,*] and Juan Zou [1,*]

1 School of Pharmacy, Guizhou University of Traditional Chinese Medicine, Guian District, Guiyang 550025, China; luojinfang66666666@163.com (J.-F.L.); wtt5840@163.com (T.-T.W.); zhaochenliang014@gzy.edu.cn (C.-L.Z.); yejianghai013@gzy.edu.cn (J.-H.Y.)
2 School of Basic Medicine, Guizhou University of Traditional Chinese Medicine, Guian District, Guiyang 550025, China
* Correspondence: hekang@gzy.edu.cn (K.H.); zoujuan466@gzy.edu.cn (J.Z.)
† These authors contributed equally to this work.

Abstract: The roots of *Astilbe grandis*, known as "Ma sang gou bang", are used as a Miao traditional medicine with anti-inflammatory and analgesic properties. However, the active components and mechanism of action of this plant remain mostly uncharacterized. The aim of this study was to identify its active components and verify their pharmacological activity. The extract of *A. grandis* root was separated using various chromatographic methods. As a result, we obtained one novel triterpenoid, named astigranlactone (**1**), which has an unusual lactone moiety formed between C-7 and C-27. Additionally, a known coumarin compound, 11-*O*-galloyl bergenin (**2**) was isolated from this plant. The structures of these two compounds were elucidated by extensive NMR experiments in conjunction with HR-ESI-MS data. To the best of our knowledge, both compounds were isolated from this species for the first time. Moreover, we tested the anti-inflammation effect of the two compounds by establishing a cellular inflammation model induced by LPS in RAW264.7 cells. The effect of different concentrations of these compounds on the activity of RAW264.7 cells was assessed using a CCK8 assay. The levels of nitric oxide (NO), tumor necrosis factor-α (TNF-α), interleukin-6 (IL-6) and interleukin-1β (IL-1β) in the supernatant of each group were evaluated using the Griess method and an enzyme-linked immunosorbent assay (ELISA). Western blot and quantitative real-time PCR (qRT-RCR) were used to measure the levels of cyclooxygenase 2 (COX-2) and nitric oxide synthase (iNOS) gene expression. Our findings revealed that these two compounds inhibited the high levels of NO, TNF-α, IL-6, IL-1β, COX-2, and iNOS (induced by LPS). Mechanistic studies demonstrated that these two compounds reduced the activation of the nuclear transcription factor-B (NF-κB) signaling pathway by inhibiting the phosphorylation of p65. Therefore, our study indicates that compounds **1** and **2** can exert a definite anti-inflammatory effect by inhibiting the NF-κB signaling pathway.

List of Contents

Table S1. ^{1}H-NMR and ^{13}C-NMR data of astigranlactone

Table S2. The primer sequences for RT-PCR

Figure S1. Key HMBC and ROESY correlations of astigranlactone

Figure S2. ^{1}H-NMR spectrum of astigranlactone

Figure S3. ^{13}C-NMR and DEPT spectra of astigranlactone

Figure S4. HSQC spectrum of astigranlactone

Figure S5. HMBC spectrum of astigranlactone

Figure S6. COSY spectrum of astigranlactone

Figure S7. ROESY spectrum of astigranlactone

Figure S8. HR-ESI-MS spectrum of astigranlactone

Figure S9. IR spectrum of astigranlactone

Table S1 ^{1}H-NMR and ^{13}C-NMR data of astigranlactone (500 MHz, $CDCl_3$, δ in ppm, J in Hz).

Position	astigranlactone	
	δ_H	δ_C
1a	1.38 (1H, m)	38.0 (t)
1b	1.72 (1H, m)	
2a	1.64 (1H, m)	23.2 (t)
2b	1.72 (1H, m)	
3	4.40 (1H, dd, 11.7, 4.1)	79.5 (d)
4	-	36.3 (s)
5	2.80 (1H, s)	59.7 (d)
6	-	206.5 (s)
7	4.18 (1H, s)	86.1 (d)
8	-	50.9 (s)
9	1.82 (1H, m)	49.8 (d)
10	-	44.8 (s)
11a	1.91 (1H, m)	22.1 (t)
11b	2.00 (1H, m)	
12	5.65 (1H, dd, 5.4, 2.0)	127.4 (d)
13	-	135.0 (s)
14	-	55.3 (s)
15a	1.48 (1H, m)	22.7 (t)
15b	1.82 (1H, m)	
16a	0.91 (1H, m)	26.4 (t)
16b	2.10 (1H, m)	
17	-	32.5 (s)
18	2.10 (1H, m)	48.6 (d)
19a	0.94 (1H, m)	42.6 (t)
19b	2.58 (1H, t, 13.8)	
20	-	30.9 (s)
21a	1.11 (1H, m)	34.5 (t)
21b	1.38 (1H, m)	
22a	1.25 (1H, m)	36.7 (t)
22b	1.48 (1H, m)	
23	0.93 (3H, s)	27.2 (q)
24	1.29 (3H, s)	16.6 (q)
25	0.97 (3H, s)	17.3 (q)
26	1.03 (3H, s)	11.3 (q)
27	-	176.7 (s)
28	0.84 (3H, s)	28.3 (q)
29	0.95 (3H, s)	33.2 (q)
30	0.89 (3H, s)	24.1 (q)

31	-	170.8 (s)
32	2.04 (3H, s)	21.3 (q)

Table S2. The primer sequences for RT-PCR

Target gene	Primer sequences
β-actin_F	5'-CGGTTCCGATGCCCTGAGGCTCTT-3'
β-actin_R	5'-CGTCACACTTCATGATGGAATTGA-3'
iNOS_F	5'-CAGCACAGGAAATGTTTCAGC-3'
iNOS_R	5'-TAGCCAGCGTACCGGATGA-3'
COX-2_F	5'-TTTGGTCTGGTGCCTGGTC-3'
COX-2_R	5'-CTGCTGGTTTGGAATAGTTGCTC-3'

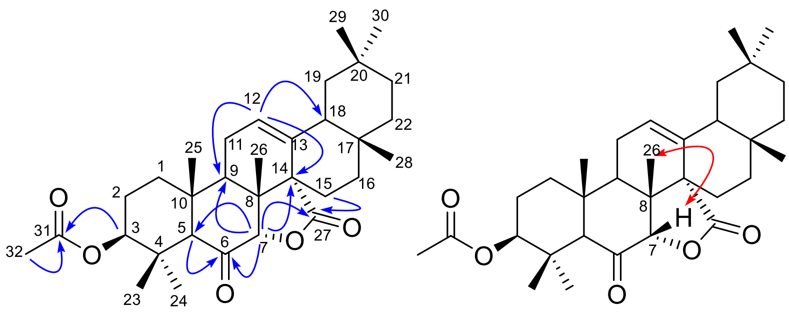

Figure S1. HMBC (→) and ROESY (↔) correlations of astigranlactone

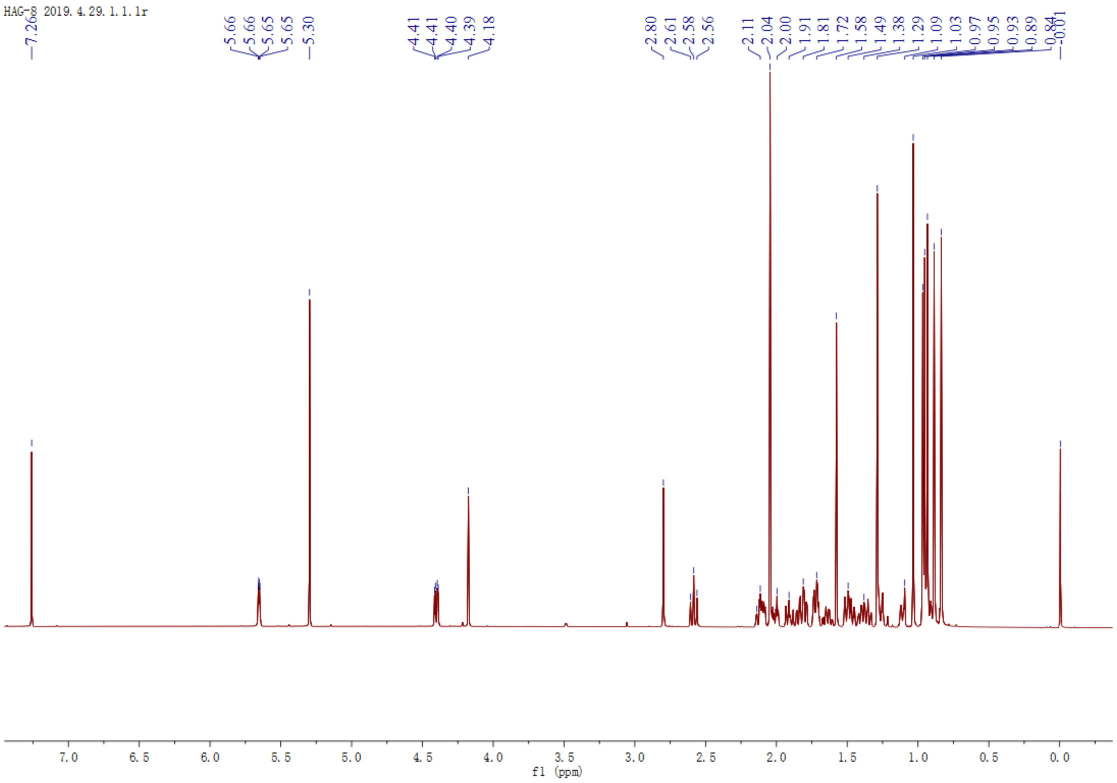

Figure S2. ^{1}H-NMR spectrum of astigranlactone

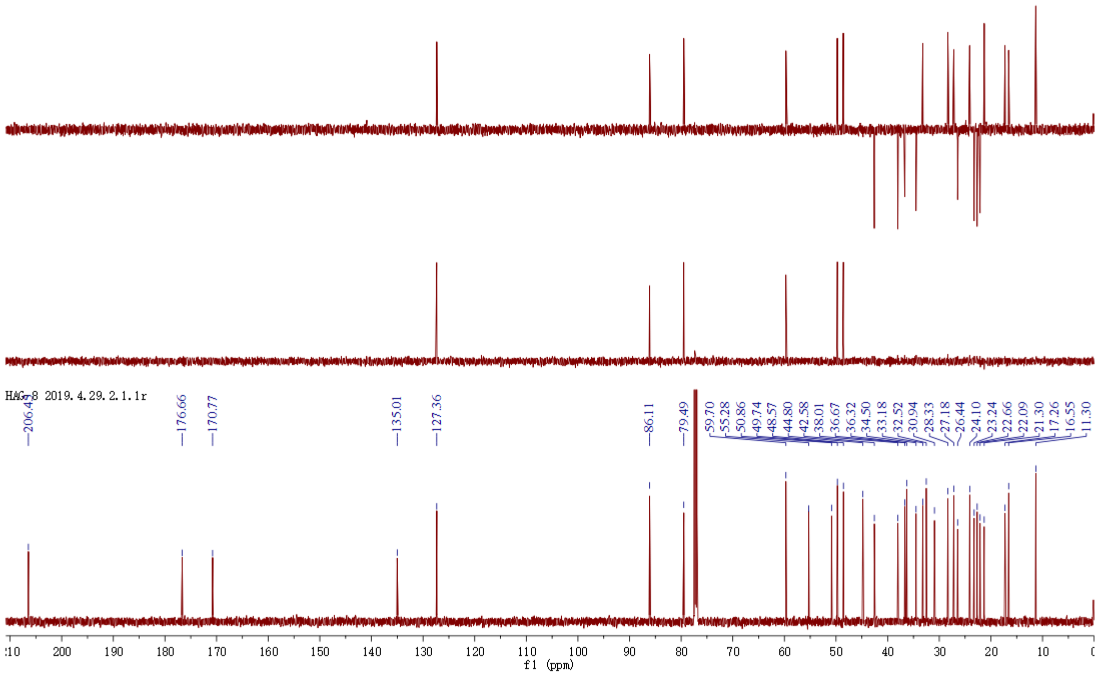

Figure S3. ^{13}C-NMR and DEPT spectra of astigranlactone

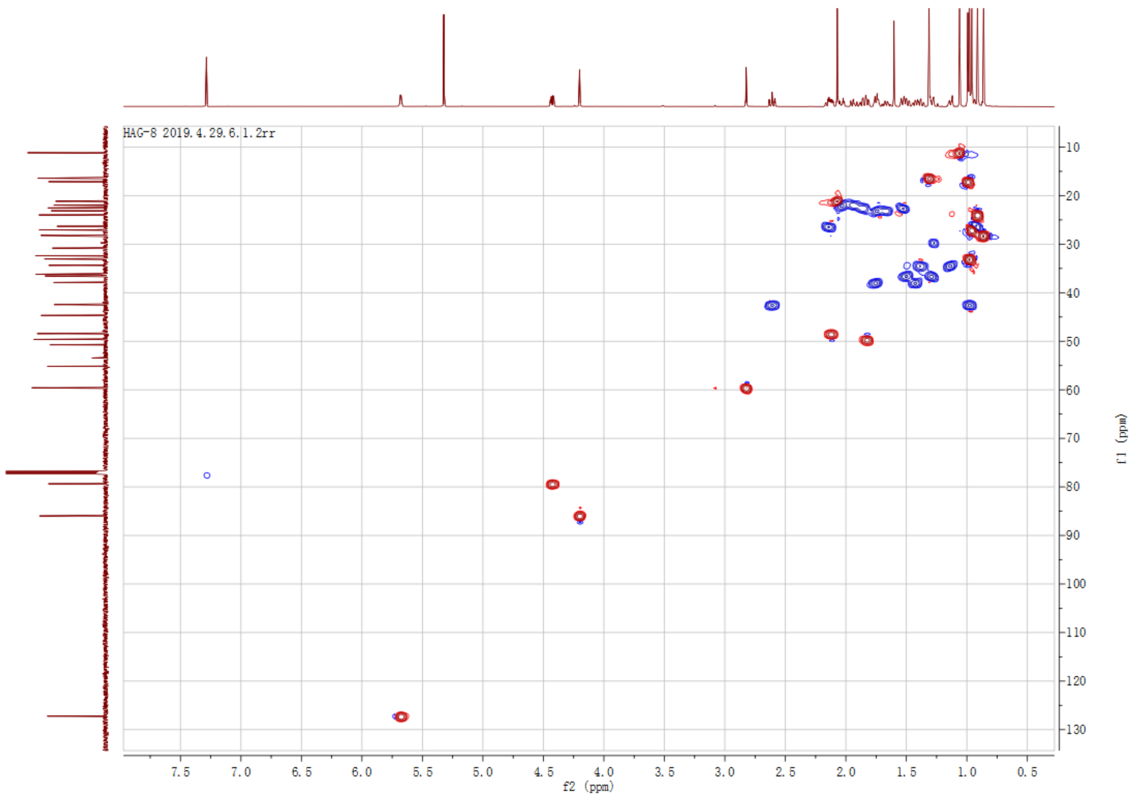

Figure S4. HMQC spectrum of astigranlactone

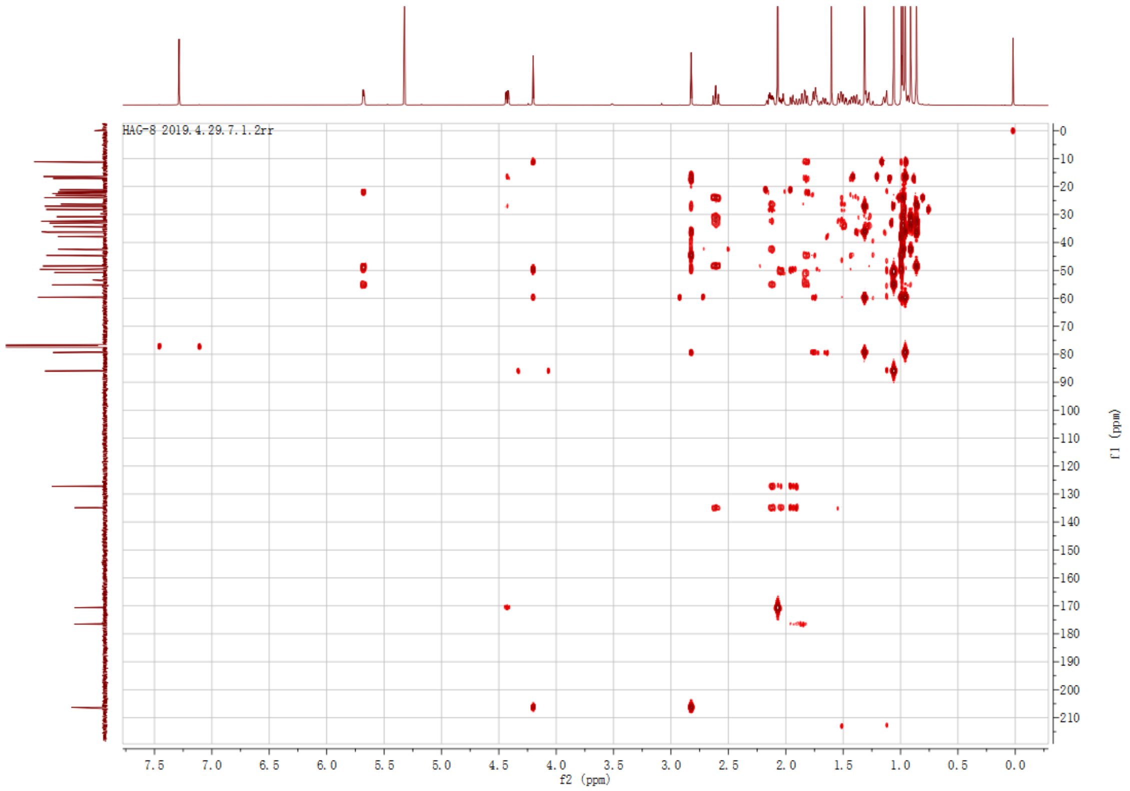

Figure S5. HMBC spectrum of astigranlactone

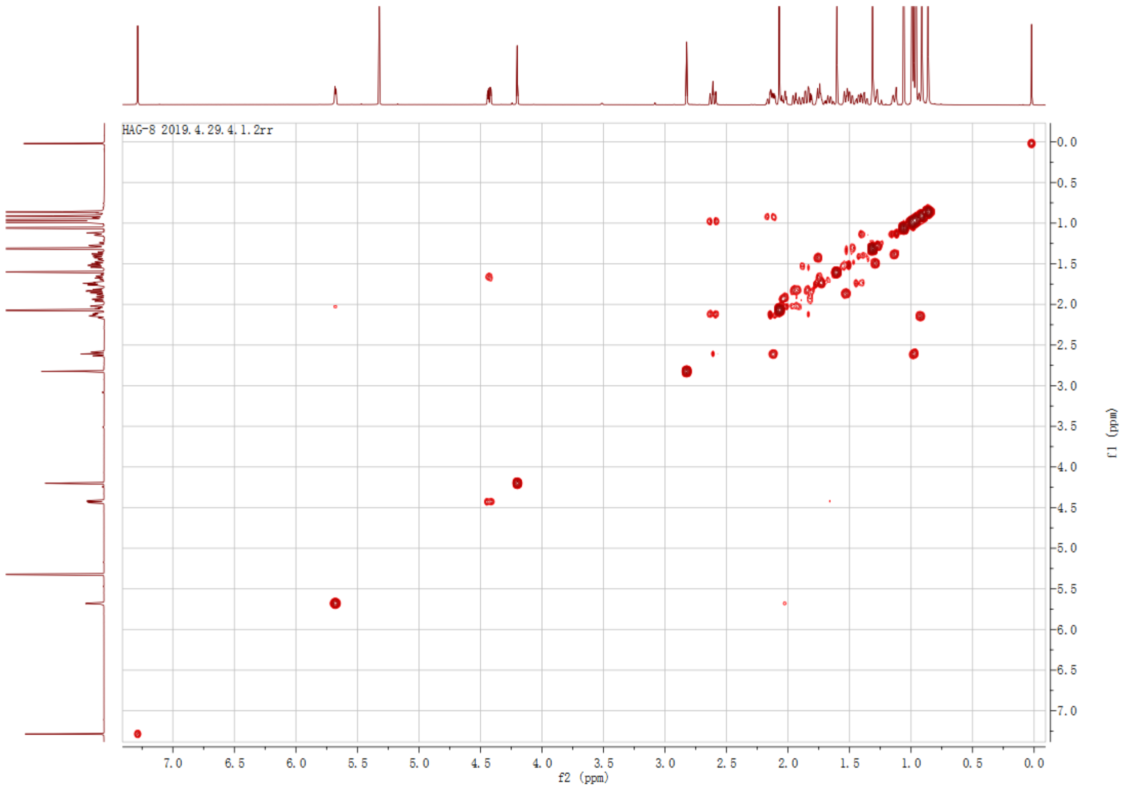

Figure S6. COSY spectrum of astigranlactone

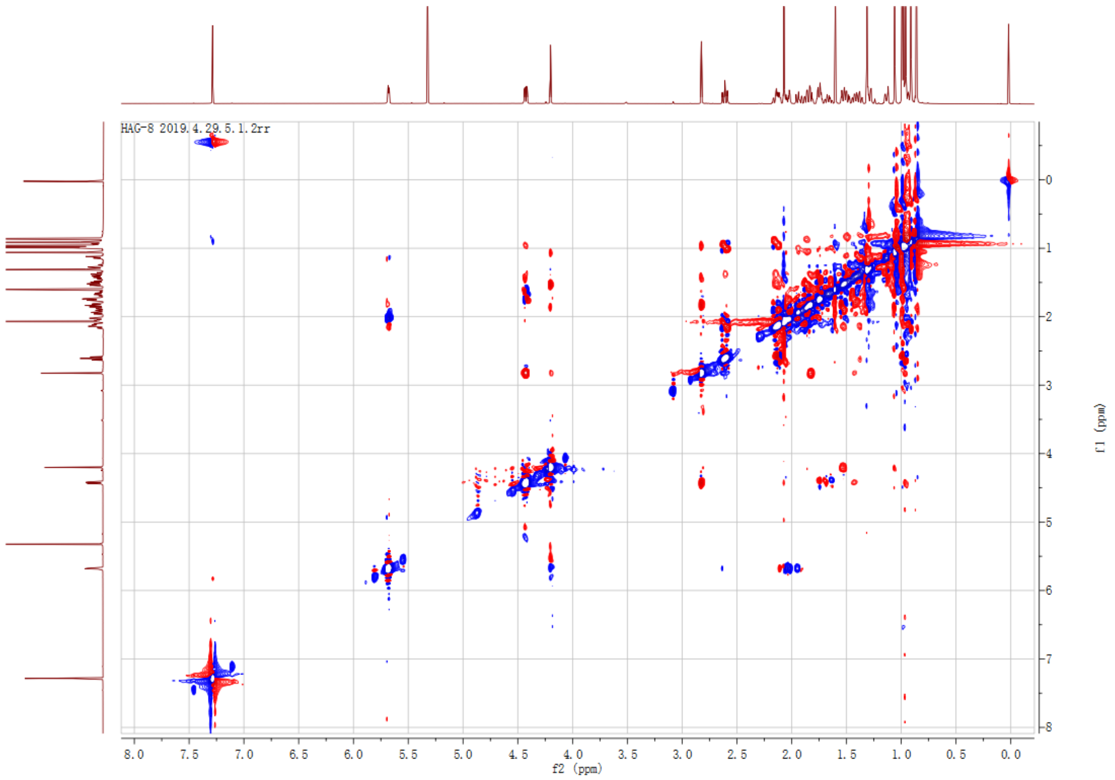

Figure S7. ROESY spectrum of astigranlactone

Qualitative Analysis Report

**Data Filename**	191017ESIA3.d	**Sample Name**	HAg-8
**Sample Type**	Sample	**Position**	
**Instrument Name**	Agilent G6230 TOF MS	**User Name**	KIB
**Acq Method**	ESI.m	**Acquired Time**	10/17/2019 3:26:47 PM
**IRM Calibration Status**	Success	**DA Method**	ESI.m
**Comment**			

Sample Group **Info.**
Acquisition SW 6200 series TOF/6500 series
Version Q-TOF B.05.01 (B5125.2)

User Spectra

Fragmentor Voltage	Collision Energy	Ionization Mode
280	0	ESI

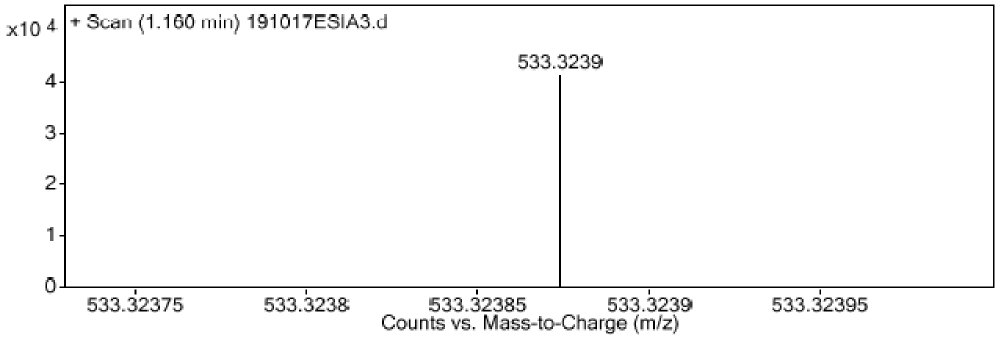

Peak List

m/z	z	Abund	Formula	Ion
533.3239	1	41335.67	C32 H46 Na O5	M+
574.3499	1	46162.97		
922.0098	1	124531.57		
1043.6573	1	95505.44		
1044.6605	1	66962.42		
1141.6555	1	64040.9		
1142.6586	1	42670.09		
1553.9919	1	161246.7		
1554.9946	1	176457.48		
1555.9971	1	94242.75		

Formula Calculator Element Limits

Element	Min	Max
C	0	200
H	0	400
O	0	10
Na	1	1

Formula Calculator Results

Formula	CalculatedMass	Mz	Diff.(mDa)	Diff. (ppm)	DBE
C32 H46 Na O5	533.3243	533.3239	0.4	0.7	9.5

Figure S8. HR-ESI-MS spectrum of astigranlactone

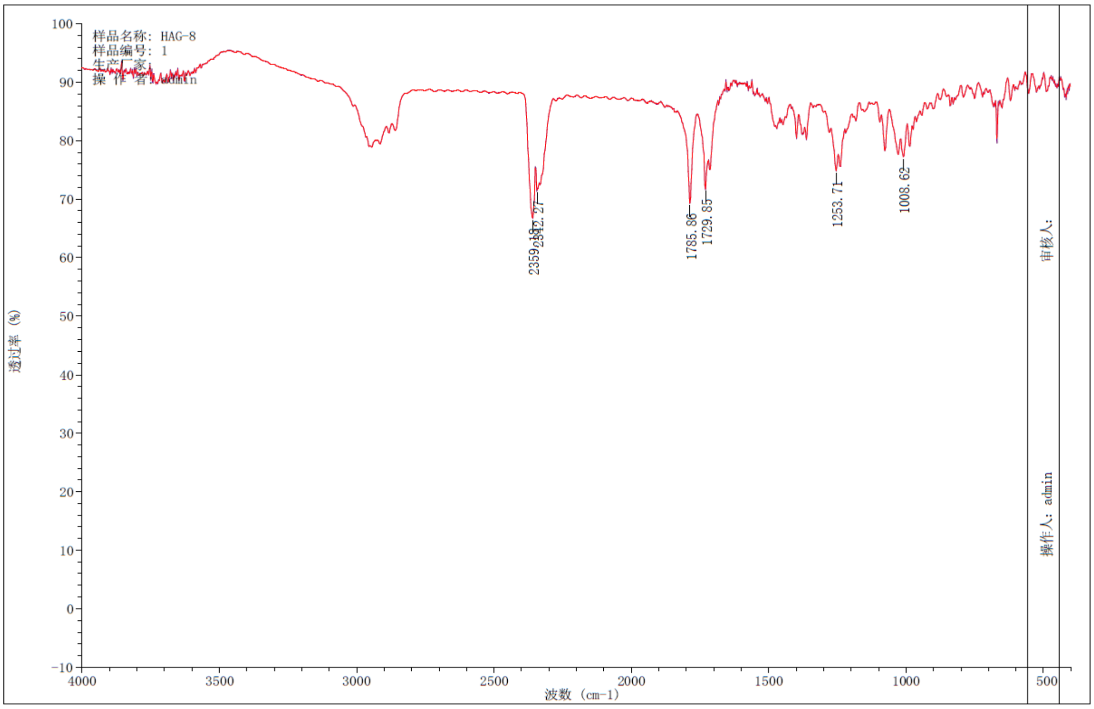

Figure S9. IR spectrum of astigranlactone
